# Tetra­kis(μ_3_-2-{[1,1-bis­(hydroxy­meth­yl)-2-oxidoeth­yl]imino­meth­yl}-6-methoxy­phenol­ato)tetra­nickel(II) tetra­hydrate

**DOI:** 10.1107/S1600536808009872

**Published:** 2008-04-16

**Authors:** Yujing Guo, Lianzhi Li, Yan Liu, Jianfang Dong, Daqi Wang

**Affiliations:** a School of Chemistry and Chemical Engineering, Liaocheng University, Shandong 252059, People’s Republic of China

## Abstract

The title complex, [Ni_4_(C_12_H_15_NO_4_)_4_]·4H_2_O, has crystal­lographic fourfold inversion symmetry, with each Ni^II^ ion coordinated in a slightly distorted square-pyramidal coordination environment and forming an Ni_4_O_4_ cubane-like core. In the crystal structure, inter­molecular O—H⋯O hydrogen bonds connect complex and water mol­ecules to form a three-dimensional network. The O atom of one of the unique hydroxy­methyl groups is disordered over two sites, with the ratio of occupancies being approximately 0.79:0.21.

## Related literature

For related literature, see: Dong, Li, Xu & Wang (2007 ▶); Dong, Li, Xu, Cui & Wang (2007 ▶); Koikawa *et al.* (2005 ▶); Mishtu *et al.* (2002 ▶); Nihei *et al.* (2003 ▶).
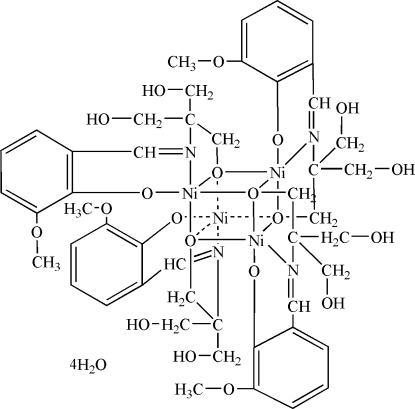


## Experimental

### 

#### Crystal data

[Ni_4_(C_12_H_15_NO_4_)_4_]·4H_2_O
*M* *_r_* = 1319.90
Tetragonal, 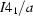

*a* = 18.754 (2) Å
*c* = 15.4395 (15) Å
*V* = 5430.3 (10) Å^3^
*Z* = 4
Mo *K*α radiation
μ = 1.45 mm^−1^
*T* = 298 (2) K
0.30 × 0.29 × 0.28 mm

#### Data collection

Bruker SMART CCD area-detector diffractometer
Absorption correction: multi-scan (*SADABS*; Sheldrick, 1996 ▶) *T*_min_ = 0.670, *T*_max_ = 0.686
11110 measured reflections
2399 independent reflections
1840 reflections with *I* > 2σ(*I*)
*R*_int_ = 0.034

#### Refinement

*R*[*F* ^2^ > 2σ(*F* ^2^)] = 0.066
*wR*(*F* ^2^) = 0.194
*S* = 1.08
2399 reflections
186 parameters
H-atom parameters constrained
Δρ_max_ = 1.23 e Å^−3^
Δρ_min_ = −0.70 e Å^−3^

### 

Data collection: *SMART* (Siemens, 1996 ▶); cell refinement: *SAINT* (Siemens, 1996 ▶); data reduction: *SAINT*; program(s) used to solve structure: *SHELXS97* (Sheldrick, 2008 ▶); program(s) used to refine structure: *SHELXL97* (Sheldrick, 2008 ▶); molecular graphics: *SHELXTL* (Sheldrick, 2008 ▶); software used to prepare material for publication: *SHELXTL*.

## Supplementary Material

Crystal structure: contains datablocks global, I. DOI: 10.1107/S1600536808009872/lh2612sup1.cif

Structure factors: contains datablocks I. DOI: 10.1107/S1600536808009872/lh2612Isup2.hkl

Additional supplementary materials:  crystallographic information; 3D view; checkCIF report

## Figures and Tables

**Table:** 

Ni1—O1	1.912 (4)
Ni1—O3	1.941 (4)
Ni1—N1	1.949 (6)
Ni1—O3^i^	1.970 (4)
Ni1—O3^ii^	2.565 (5)

**Table:** 

O1—Ni1—O3	172.2 (2)
O1—Ni1—N1	94.3 (2)
O3—Ni1—N1	84.1 (2)
O1—Ni1—O3^i^	94.57 (19)
O3—Ni1—O3^i^	88.47 (19)
N1—Ni1—O3^i^	166.1 (2)
O1—Ni1—O3^ii^	94.23 (17)
O3—Ni1—O3^ii^	79.80 (17)
N1—Ni1—O3^ii^	117.2 (2)
O3^i^—Ni1—O3^ii^	72.63 (17)

Symmetry codes: (i) 
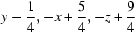
; (ii) 
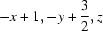
.

**Table 2:** Hydrogen-bond geometry (Å, °)

*D*—H⋯*A*	*D*—H	H⋯*A*	*D*⋯*A*	*D*—H⋯*A*
O5—H5⋯N1	0.82	2.58	2.988 (9)	112
O4—H4⋯O6^iii^	0.82	1.94	2.714 (8)	157
O4′—H4′⋯O6^iii^	0.82	1.96	2.68 (3)	148
O6—H6*A*⋯O1^iv^	0.85	1.95	2.803 (7)	180
O6—H6*B*⋯O4^v^	0.85	2.04	2.892 (9)	180

Symmetry codes: (iii) 
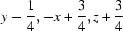
; (iv) 
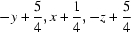
; (v) 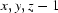.

**Fig. 1.**
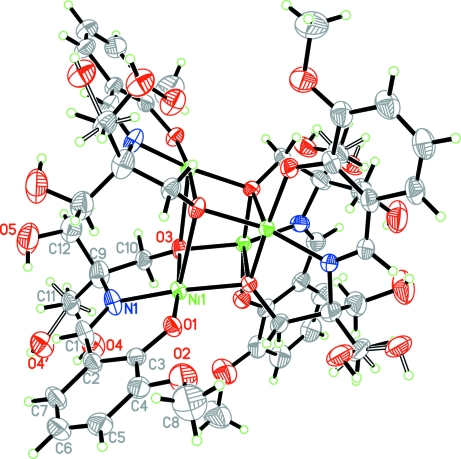
The structure of the title compound, showing 30% probability displacement ellipsoids and the atom-numbering scheme. The water solvent molecules are not shown. Open bonds indicate disordered atoms and only the assymetric unit is labelled.

**Fig. 2.**
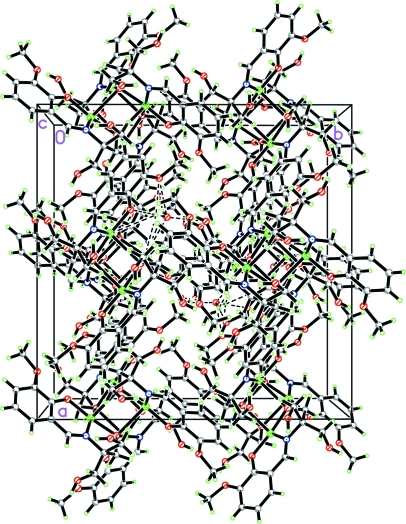
Part of the crystal structure with hydrogen bonds shown as dashed lines. The disorder is not shown.

## supplementary crystallographic information

### Comment

The chemistry of transition metal ion complexes of hydroxy (aryl-OH and alkyl-OH) rich molecules containing imine/amine group is important in the biomimetic studies of metalloproteins (Mishtu *et al.*, 2002). Polynuclear metal complexes with tridentate ligand containing hydroxyl groups as terminal coordinating atoms have been reported and have attracted much attention (Nihei *et al.*, 2003).

A few structurally characterized multinuclear complexes containing Schiff base ligands has been reported( e.g. Dong, Li, Xu & Wang (2007); Dong, Li, Xu, Cui & Wang (2007); Nihei *et al.*, 2003). Herein, we report the synthesis and crystal structure of a novel tetranickel(II) complex with a tridentate Schiff base ligand derived from the condensation of *o*-vanillin and trihydroxymethylaminomethane.

The title compound contains a tetranuclear cubane core based on an approximately cubic array of alternating nickel and oxygen atoms (Fig.1). Each Ni^II^ ion is in a distorted square-pyramidal coordination environment with one nitrogen and two oxygen atoms from one Schiff base ligand and two oxygen atoms from the symmetry related units of the cubane core. The Ni atom deviates from the basal plane (formed by O1, N1, O3 and O3^i^, symmetry code (i) *y* - 7/4, -*x* + 3/4, -*z* + 7/4) by 0.1299 (33) Å, with a significantly longer Ni—O_apical_ bond distance (Table 1). In the molecular structure, the Ni—Ni distances (3.472 (4) Å, 3.182 (3) Å) are longer than some reported values (Koikawa *et al.*, 2005). In addition, there are four H_2_O solvent molecules, which are involved in intermolecular O-H···O hydrogen bonds (Fig. 2, Table 2) which stabilize the crystal atructure along with van der Waals forces.

### Experimental

Trihydroxymethylaminomethane(1 mmol, 121.14 mg) was dissolved in hot methanol (10 ml) and added successively to a methanol solution(3 ml) of *o*-vanillin (1 mmol, 152.15 mg). The mixture was then stirred at 323 K for 2 h. Subsequently, an aqueous solution(2 ml) of nickel chlorate hexahydrate(1 mmol, 237.66 mg) was added dropwise and stirred for another 5 h. The solution was held at room temperature for ten days, whereupon green blocky crystals suitable for X-ray diffraction were obtained.

### Refinement

Difference Fourier maps revealed that one of the hydroxymethyl group is distorted over two sites. The subsequent refinement of their occupancies gave the value of 0.791 (3) and 0.209 (3), respectively. All the H atoms were placed in geometrically calculated positions (C—H = 0.93 - 0.97 Å, O—H = 0.82 Å) and allowed to ride on their respective parent atoms, with *U*_iso_(H) = 1.2*U*_eq_(C) or 1.5*U*_eq_(C_methyl_).

### Crystal data

**Table:**

[Ni_4_(C_12_H_15_NO_4_)_4_]·4H_2_O	*Z* = 4
*M**_r_* = 1319.90	*F*_000_ = 2752
Tetragonal, *I*4_1_/*a*	*D*_x_ = 1.614 Mg m^−^^3^
Hall symbol: -I 4ad	Mo *K*α radiation λ = 0.71073 Å
*a* = 18.754 (2) Å	Cell parameters from 3768 reflections
*b* = 18.754 (2) Å	θ = 2.2–25.2º
*c* = 15.4395 (15) Å	µ = 1.45 mm^−^^1^
α = 90º	*T* = 298 (2) K
β = 90º	Block, green
γ = 90º	0.30 × 0.29 × 0.28 mm
*V* = 5430.3 (10) Å^3^	

### Data collection

**Table:**

Bruker SMART CCD area-detector diffractometer	2399 independent reflections
Radiation source: fine-focus sealed tube	1840 reflections with *I* > 2σ(*I*)
Monochromator: graphite	*R*_int_ = 0.034
*T* = 298(2) K	θ_max_ = 25.0º
φ and ω scans	θ_min_ = 1.7º
Absorption correction: multi-scan(SADABS; Sheldrick, 1996)	*h* = −22→22
*T*_min_ = 0.670, *T*_max_ = 0.686	*k* = −22→15
11110 measured reflections	*l* = −9→18

### Refinement

**Table:**

Refinement on *F*^2^	Secondary atom site location: difference Fourier map
Least-squares matrix: full	Hydrogen site location: inferred from neighbouring sites
*R*[*F*^2^ > 2σ(*F*^2^)] = 0.066	H-atom parameters constrained
*wR*(*F*^2^) = 0.194	*w* = 1/[σ^2^(*F*_o_^2^) + (0.0801*P*)^2^ + 60.7787*P*] where *P* = (*F*_o_^2^ + 2*F*_c_^2^)/3
*S* = 1.08	(Δ/σ)_max_ = 0.001
2399 reflections	Δρ_max_ = 1.23 e Å^−^^3^
186 parameters	Δρ_min_ = −0.70 e Å^−^^3^
Primary atom site location: structure-invariant direct methods	Extinction correction: none

### Special details

**Table:**

Geometry. All e.s.d.'s (except the e.s.d. in the dihedral angle between two l.s. planes) are estimated using the full covariance matrix. The cell e.s.d.'s are taken into account individually in the estimation of e.s.d.'s in distances, angles and torsion angles; correlations between e.s.d.'s in cell parameters are only used when they are defined by crystal symmetry. An approximate (isotropic) treatment of cell e.s.d.'s is used for estimating e.s.d.'s involving l.s. planes.
Refinement. Refinement of *F*^2^ against ALL reflections. The weighted *R*-factor *wR* and goodness of fit *S* are based on *F*^2^, conventional *R*-factors *R* are based on *F*, with *F* set to zero for negative *F*^2^. The threshold expression of *F*^2^ > σ(*F*^2^) is used only for calculating *R*-factors(gt) *etc*. and is not relevant to the choice of reflections for refinement. *R*-factors based on *F*^2^ are statistically about twice as large as those based on *F*, and *R*- factors based on ALL data will be even larger.

### Fractional atomic coordinates and isotropic or equivalent isotropic displacement parameters (Å^2^)

**Table:**

	*x*	*y*	*z*	*U*_iso_*/*U*_eq_	Occ. (<1)
Ni1	0.40964 (4)	0.72996 (4)	1.05945 (5)	0.0374 (3)	
N1	0.3971 (4)	0.6819 (3)	0.9486 (4)	0.0575 (16)	
O1	0.3327 (2)	0.7952 (2)	1.0412 (3)	0.0480 (11)	
O2	0.2316 (3)	0.8868 (4)	1.0480 (5)	0.097 (2)	
O3	0.4952 (2)	0.6720 (2)	1.0675 (3)	0.0435 (10)	
O4	0.3704 (5)	0.5372 (4)	0.9346 (5)	0.079 (2)	0.791 (10)
H4	0.3533	0.5023	0.9103	0.119*	0.791 (10)
O4'	0.3587 (16)	0.5547 (15)	0.836 (2)	0.079 (2)	0.209 (10)
H4'	0.3562	0.5134	0.8190	0.119*	0.209 (10)
O5	0.4819 (5)	0.6865 (5)	0.7848 (5)	0.120 (3)	
H5	0.4383	0.6853	0.7893	0.181*	
O6	0.3380 (3)	0.6042 (3)	0.0986 (4)	0.0729 (16)	
H6A	0.3733	0.5978	0.1322	0.088*	
H6B	0.3472	0.5846	0.0503	0.088*	
C1	0.3427 (4)	0.6889 (4)	0.9010 (5)	0.061 (2)	
H1	0.3392	0.6586	0.8535	0.073*	
C2	0.2852 (4)	0.7394 (4)	0.9132 (5)	0.0529 (18)	
C3	0.2839 (3)	0.7900 (4)	0.9815 (4)	0.0476 (16)	
C4	0.2269 (4)	0.8386 (5)	0.9823 (5)	0.067 (2)	
C5	0.1712 (5)	0.8344 (6)	0.9218 (6)	0.078 (3)	
H5A	0.1334	0.8664	0.9245	0.094*	
C6	0.1727 (5)	0.7834 (6)	0.8595 (6)	0.078 (3)	
H6	0.1354	0.7800	0.8201	0.094*	
C7	0.2279 (4)	0.7377 (5)	0.8544 (6)	0.068 (2)	
H7	0.2281	0.7038	0.8104	0.081*	
C8	0.1829 (7)	0.9466 (7)	1.0493 (9)	0.130 (5)	
H8A	0.1797	0.9668	0.9923	0.195*	
H8B	0.2001	0.9820	1.0891	0.195*	
H8C	0.1366	0.9306	1.0675	0.195*	
C9	0.4572 (5)	0.6290 (5)	0.9262 (6)	0.075 (3)	
C10	0.4932 (4)	0.6144 (4)	1.0099 (4)	0.0523 (17)	
H10A	0.4691	0.5749	1.0380	0.063*	
H10B	0.5418	0.5995	0.9981	0.063*	
C11	0.4273 (5)	0.5654 (5)	0.8832 (6)	0.073 (2)	
H11A	0.4096	0.5781	0.8262	0.088*	0.791 (10)
H11B	0.4642	0.5296	0.8762	0.088*	0.791 (10)
H11C	0.4635	0.5509	0.8420	0.088*	0.209 (10)
H11D	0.4276	0.5290	0.9279	0.088*	0.209 (10)
C12	0.5136 (5)	0.6631 (6)	0.8640 (6)	0.085 (3)	
H12A	0.5359	0.7033	0.8927	0.101*	
H12B	0.5504	0.6283	0.8514	0.101*	

### Atomic displacement parameters (Å^2^)

**Table:**

	*U*^11^	*U*^22^	*U*^33^	*U*^12^	*U*^13^	*U*^23^
Ni1	0.0427 (5)	0.0358 (5)	0.0337 (5)	0.0002 (3)	−0.0072 (3)	−0.0007 (3)
N1	0.080 (4)	0.046 (3)	0.046 (3)	0.015 (3)	−0.022 (3)	−0.009 (3)
O1	0.045 (3)	0.055 (3)	0.043 (2)	0.008 (2)	−0.009 (2)	−0.005 (2)
O2	0.070 (4)	0.125 (6)	0.095 (5)	0.048 (4)	−0.010 (4)	−0.019 (4)
O3	0.056 (3)	0.039 (2)	0.036 (2)	0.010 (2)	−0.008 (2)	−0.0013 (19)
O4	0.094 (6)	0.059 (4)	0.086 (5)	0.007 (4)	−0.005 (5)	−0.030 (4)
O4'	0.094 (6)	0.059 (4)	0.086 (5)	0.007 (4)	−0.005 (5)	−0.030 (4)
O5	0.111 (6)	0.189 (8)	0.061 (4)	0.038 (6)	0.005 (4)	0.011 (5)
O6	0.053 (3)	0.096 (4)	0.070 (4)	−0.001 (3)	0.011 (3)	−0.003 (3)
C1	0.079 (5)	0.056 (4)	0.048 (4)	0.006 (4)	−0.022 (4)	−0.007 (3)
C2	0.055 (4)	0.059 (4)	0.045 (4)	−0.010 (3)	−0.013 (3)	0.007 (3)
C3	0.039 (4)	0.059 (4)	0.044 (4)	−0.001 (3)	−0.002 (3)	0.014 (3)
C4	0.049 (4)	0.093 (6)	0.058 (5)	0.010 (4)	−0.001 (4)	0.002 (5)
C5	0.050 (5)	0.110 (8)	0.075 (6)	0.014 (5)	−0.004 (4)	0.010 (6)
C6	0.060 (5)	0.106 (7)	0.069 (6)	−0.008 (5)	−0.018 (4)	0.007 (5)
C7	0.064 (5)	0.081 (6)	0.058 (5)	−0.011 (4)	−0.025 (4)	0.005 (4)
C8	0.105 (9)	0.148 (12)	0.137 (12)	0.064 (9)	−0.013 (8)	−0.026 (9)
C9	0.088 (6)	0.076 (6)	0.061 (5)	0.035 (5)	−0.005 (5)	−0.016 (4)
C10	0.054 (4)	0.060 (4)	0.043 (4)	0.010 (3)	−0.001 (3)	−0.013 (3)
C11	0.081 (6)	0.076 (6)	0.061 (5)	0.016 (5)	−0.007 (5)	−0.031 (5)
C12	0.085 (7)	0.104 (8)	0.065 (6)	0.024 (6)	0.001 (5)	−0.002 (5)

### Geometric parameters (Å, °)

**Table:**

Ni1—O1	1.912 (4)	C2—C7	1.408 (10)
Ni1—O3	1.941 (4)	C2—C3	1.419 (10)
Ni1—N1	1.949 (6)	C3—C4	1.405 (11)
Ni1—O3^i^	1.970 (4)	C4—C5	1.403 (12)
Ni1—O3^ii^	2.565 (5)	C5—C6	1.357 (13)
N1—C1	1.265 (9)	C5—H5A	0.9300
N1—C9	1.541 (10)	C6—C7	1.345 (13)
O1—C3	1.303 (8)	C6—H6	0.9300
O2—C4	1.362 (11)	C7—H7	0.9300
O2—C8	1.446 (12)	C8—H8A	0.9600
O3—C10	1.400 (8)	C8—H8B	0.9600
O3—Ni1^iii^	1.970 (4)	C8—H8C	0.9600
O4—C11	1.430 (12)	C9—C11	1.475 (13)
O4—H4	0.8200	C9—C10	1.484 (11)
O4—H11D	1.0883	C9—C12	1.565 (14)
O4'—C11	1.49 (3)	C10—H10A	0.9700
O4'—H4'	0.8200	C10—H10B	0.9700
O5—C12	1.429 (12)	C11—H11A	0.9700
O5—H5	0.8200	C11—H11B	0.9700
O6—H6A	0.8500	C11—H11C	0.9698
O6—H6B	0.8499	C11—H11D	0.9699
C1—C2	1.447 (11)	C12—H12A	0.9700
C1—H1	0.9300	C12—H12B	0.9700
			
O1—Ni1—O3	172.2 (2)	O2—C8—H8A	109.5
O1—Ni1—N1	94.3 (2)	O2—C8—H8B	109.5
O3—Ni1—N1	84.1 (2)	H8A—C8—H8B	109.5
O1—Ni1—O3^i^	94.57 (19)	O2—C8—H8C	109.5
O3—Ni1—O3^i^	88.47 (19)	H8A—C8—H8C	109.5
N1—Ni1—O3^i^	166.1 (2)	H8B—C8—H8C	109.5
O1—Ni1—O3^ii^	94.23 (17)	C11—C9—C10	114.6 (8)
O3—Ni1—O3^ii^	79.80 (17)	C11—C9—N1	110.2 (8)
N1—Ni1—O3^ii^	117.2 (2)	C10—C9—N1	104.9 (6)
O3^i^—Ni1—O3^ii^	72.63 (17)	C11—C9—C12	108.1 (8)
C1—N1—C9	121.7 (6)	C10—C9—C12	107.5 (8)
C1—N1—Ni1	124.1 (6)	N1—C9—C12	111.6 (7)
C9—N1—Ni1	114.0 (5)	O3—C10—C9	115.0 (6)
C3—O1—Ni1	125.9 (4)	O3—C10—H10A	108.5
C4—O2—C8	119.0 (8)	C9—C10—H10A	108.5
C10—O3—Ni1	111.7 (4)	O3—C10—H10B	108.5
C10—O3—Ni1^iii^	121.9 (4)	C9—C10—H10B	108.5
Ni1—O3—Ni1^iii^	108.9 (2)	H10A—C10—H10B	107.5
C11—O4—H4	109.5	O4—C11—C9	109.4 (7)
H4—O4—H11D	103.2	O4—C11—O4'	65.0 (13)
C11—O4'—H4'	109.5	C9—C11—O4'	130.9 (13)
C12—O5—H5	109.5	O4—C11—H11A	109.8
H6A—O6—H6B	108.4	C9—C11—H11A	109.8
N1—C1—C2	126.4 (7)	O4'—C11—H11A	45.3
N1—C1—H1	116.8	O4—C11—H11B	109.8
C2—C1—H1	116.8	C9—C11—H11B	109.8
C7—C2—C3	118.8 (7)	O4'—C11—H11B	117.9
C7—C2—C1	118.0 (7)	H11A—C11—H11B	108.2
C3—C2—C1	123.1 (6)	O4—C11—H11C	141.6
O1—C3—C4	118.7 (7)	C9—C11—H11C	104.8
O1—C3—C2	124.4 (6)	O4'—C11—H11C	104.3
C4—C3—C2	116.9 (7)	H11A—C11—H11C	73.3
O2—C4—C5	125.6 (8)	O4—C11—H11D	49.5
O2—C4—C3	112.8 (7)	C9—C11—H11D	104.3
C5—C4—C3	121.6 (9)	O4'—C11—H11D	104.9
C6—C5—C4	119.7 (9)	H11A—C11—H11D	145.1
C6—C5—H5A	120.1	H11B—C11—H11D	65.7
C4—C5—H5A	120.1	H11C—C11—H11D	105.4
C7—C6—C5	120.5 (8)	O5—C12—C9	111.7 (8)
C7—C6—H6	119.8	O5—C12—H12A	109.3
C5—C6—H6	119.8	C9—C12—H12A	109.3
C6—C7—C2	122.3 (9)	O5—C12—H12B	109.3
C6—C7—H7	118.9	C9—C12—H12B	109.3
C2—C7—H7	118.9	H12A—C12—H12B	107.9
			
C1—C2—C3—C4	176.2 (7)		

Symmetry codes: (i) *y*−1/4, −*x*+5/4, −*z*+9/4; (ii) −*x*+1, −*y*+3/2, *z*; (iii) −*y*+5/4, *x*+1/4, −*z*+9/4.

### Hydrogen-bond geometry (Å, °)

**Table:**

*D*—H···*A*	*D*—H	H···*A*	*D*···*A*	*D*—H···*A*
O5—H5···N1	0.82	2.58	2.988 (9)	112
O4—H4···O6^iv^	0.82	1.94	2.714 (8)	157
O4'—H4'···O6^iv^	0.82	1.96	2.68 (3)	148
O6—H6A···O1^v^	0.85	1.95	2.803 (7)	180
O6—H6B···O4^vi^	0.85	2.04	2.892 (9)	180

Symmetry codes: (iv) *y*−1/4, −*x*+3/4, *z*+3/4; (v) −*y*+5/4, *x*+1/4, −*z*+5/4; (vi) *x*, *y*, *z*−1.
